# New Mixture

**Published:** 1879-11-20

**Authors:** J. W. Collins


### *A NEW MIXTURE.*

I have been using for the past six years a tonic and digestive preparation that I find of considerable value, and I think that the profession should be made aware of its merits through your journal. I usually prescribe it under the name of co-phosphate powders. Its composition and mode of preparation is as follows:

℞. Ferri phos............................

Sodii phos......... ...................

Calcis phos............................aa ℥ ss.

Sach. lactis...........................℥ i.

Expose the soda to a gentle heat in a porcelain vessel until the water of crystalization is driven off, taking care to not continue the heat until it becomes caustic, having the other powders perfectly dry. Mix the whole and triturate thoroughly. Dose, from ten to twenty grains three times every day.

This preparation is pleasant and almost tasteless, and is well borne by the most delicate stomach. I have used it in almost every condition of malnutrition and general debility, and notably in incipient turberculosis, chronic bronchitis attended with emaciation. And, also, in those cases of malnutrition so common in chronic uterine diseases, hysteria and various forms of neuralgia accompanied by indigestion. In this class of cases I have found no better tonic and promoter of digestion. This is the verdict of such of my professional brethren as have given it a trial. I think its greatest efficacy is found in that unfortunate class of cases which come under the care of almost every physician in general practice; the early stages of tuberculosis, in which the much prescribed cod liver oil is so illy borne, and often inadmissible, where the patient is unable to take a sufficient quantity of the oil to arrest rapid tissue waste. Here I would most strongly urge its persistent administration. I think its mode of action is two-fold—direct and indirect. It certainly furnishes directly to the tissues iron, lime and phosphorous; and indirectly, it stimulates digestion and aids assimilation of food in its various forms. I have now under observation several cases in which I have exhibited it for tubercular disease. Two of these cases had, five years ago, unmistakable physical signs of small cavities and softening of lung tissue fully developed with emaciation and hectic fever, and after its daily use for from eight to ten months, both of them are now apparently restored to health; and I have rarely failed to observe improvement in cases treated in early stages of pulmonary tubercle. I am sure it is more reliable than cod liver oil in any of its many combinations. I also use it in certain stages of bowel and digestive troubles of children. It may be exhibited in conjunction with cod liver oil, by giving before meals and the oil after. It seems to render the oil less offensive to the alimentary canal and more easily assimilated. However, since I have learned its great value and superiority to the oil, I rarely give the latter.—*Dr. J. W. Collins, M. D., in Medical Herald.*

